# Evaluating the Impact of Different Sequence Databases on Metaproteome Analysis: Insights from a Lab-Assembled Microbial Mixture

**DOI:** 10.1371/journal.pone.0082981

**Published:** 2013-12-09

**Authors:** Alessandro Tanca, Antonio Palomba, Massimo Deligios, Tiziana Cubeddu, Cristina Fraumene, Grazia Biosa, Daniela Pagnozzi, Maria Filippa Addis, Sergio Uzzau

**Affiliations:** 1 Porto Conte Ricerche Srl, Tramariglio, Alghero, Italy; 2 Dipartimento di Scienze Biomediche, Università di Sassari, Sassari, Italy; UGent/VIB, Belgium

## Abstract

Metaproteomics enables the investigation of the protein repertoire expressed by complex microbial communities. However, to unleash its full potential, refinements in bioinformatic approaches for data analysis are still needed. In this context, sequence databases selection represents a major challenge.

This work assessed the impact of different databases in metaproteomic investigations by using a mock microbial mixture including nine diverse bacterial and eukaryotic species, which was subjected to shotgun metaproteomic analysis. Then, both the microbial mixture and the single microorganisms were subjected to next generation sequencing to obtain experimental metagenomic- and genomic-derived databases, which were used along with public databases (namely, NCBI, UniProtKB/SwissProt and UniProtKB/TrEMBL, parsed at different taxonomic levels) to analyze the metaproteomic dataset. First, a quantitative comparison in terms of number and overlap of peptide identifications was carried out among all databases. As a result, only 35% of peptides were common to all database classes; moreover, genus/species-specific databases provided up to 17% more identifications compared to databases with generic taxonomy, while the metagenomic database enabled a slight increment in respect to public databases. Then, database behavior in terms of false discovery rate and peptide degeneracy was critically evaluated. Public databases with generic taxonomy exhibited a markedly different trend compared to the counterparts. Finally, the reliability of taxonomic attribution according to the lowest common ancestor approach (using MEGAN and Unipept software) was assessed. The level of misassignments varied among the different databases, and specific thresholds based on the number of taxon-specific peptides were established to minimize false positives. This study confirms that database selection has a significant impact in metaproteomics, and provides critical indications for improving depth and reliability of metaproteomic results. Specifically, the use of iterative searches and of suitable filters for taxonomic assignments is proposed with the aim of increasing coverage and trustworthiness of metaproteomic data.

## Introduction

The interest in microbial communities has grown enormously in the last decade, due to their relevance in numerous fields spanning microbial ecology, agriculture, waste treatment, bioremediation, renewable energy production, as well as for their importance to human and animal health [1], [2], . A significant boost to the study of complex microbial communities has been provided by the latest advances in metagenomic techniques, which have allowed researchers to study a microbial population in its natural *milieu* according to a holistic approach, and therefore to gather information on interactions occurring among microorganisms and with their environment [10], [11], [12], [13]. Specifically, 16S (and 18S for eukaryotic species) rRNA gene and whole metagenome sequencing approaches can provide a snapshot of the entire community complexity in terms of taxonomic composition and genetic potential, respectively. However, expression data are required in order to gain information on the pathways that are actively functioning in a community, and on how expression of specific proteins can change according to time, location, or environmental stimuli [14]. In this respect, metaproteomics has the ability to identify and quantify the protein repertoire collectively expressed by microbes colonizing a given environment [15], [16], [17], [18].

Yet, the metaproteomic characterization of a microbial community poses several challenges, particularly concerning data analysis and interpretation, as recently reviewed [19], [20]. Two major issues affect metaproteome analysis: first, genome sequence data might be unavailable for most of the species of the microbial community under study, considerably reducing the chances for a correct matching between the experimental spectra and the theoretical spectra; second, a typical environmental sample contains thousands of proteins belonging to up to thousands of different microbial species, often having a high level of homology, making both peptide-to-protein and peptide-to-taxa assignments a tremendous task.

In this context, the selection of proper protein databases (DBs) represents an extremely critical step, especially when dealing with poorly characterized microbiomes. When a novel microbial community is subjected to metaproteome analysis, without further genomic investigation, publicly available DBs have to be used for peptide/protein identification, at least for a preliminary analysis. Protein DBs can be generally distinguished into non-manually annotated (with plenty of information, but huge dimensions, and thus very high computing times, such as NCBI and TrEMBL) and manually curated sequences (as SwissProt, with inverse pros and cons when compared to the non-annotated ones) [21], [22]. However, in spite of the great efforts made in the last years by genome scientists, most uncultivable species have not been sequenced yet, and therefore are not available in the public resources. In this case, cross-species identification can occur when genome sequences of closely related species, with large sequence homology regions, are available [23]. Unlike ‘classical’ DNA sequence homology search, in proteomics even slight differences in amino acid sequences lead to significant variations in peptide masses, making the proteomic characterization of unsequenced organisms extremely difficult. A possible alternative is using *de novo* sequencing, in which amino acid sequences are deduced directly from fragmentation spectra, without the need for a protein DB, followed by BLAST search for identification of candidate homologous proteins [24], [25]. However, manual inspection of spectra is often required due to the error-prone nature of *de novo* sequencing, and very high quality data are necessary for achieving reliable results [26].

The integration of metagenomics and metaproteomics holds promise to address the above issues, as described by an increasing number of publications in the very recent past [27], [28], [29], [30], [31], [32], [33]. Currently, such integration may occur at different levels (ordered by increasing complexity): i) using 16S (and/or18S) rRNA gene sequencing information to assemble a customized DB (also named ‘pseudo-metagenome’) restricted to the taxa which have been (or are expected to be) found within the microbiome under study, saving up analysis time and minimizing species misassignments [27], [34]; ii) using translated and annotated metagenome sequences as protein DB, ideally generated from the same sample being analyzed with metaproteomics (a so-called ‘matched’ metagenome), but also retrieved from public metagenome archives, which are expected to impressively grow in the years to come [32], [35], [36]; iii) isolating further reference strains from the microbiome under study and performing individual genome sequencing, on the basis on a labor-intensive, in-depth approach recently referred to as ‘microbial culturomics’ [37]. Furthermore, according to a proteogenomic (*sensu stricto*) approach, metagenomic and genomic sequences can also be translated in all six reading frames (six-frame translation, 6FT), with the purpose of minimizing the inherent biases derived from gene prediction methods [38], [39]. However, metagenome-derived DBs may suffer from technical issues in DNA extraction, affecting species with lower abundance or higher resistance to lysis, as well as from bioinformatic issues in sequence assembly and annotation. To the best of our knowledge, no reports have been described so far critically comparing the metaproteomic data which can be obtained using different types of publicly available and matched metagenome-derived DBs.

Interestingly, each of the above mentioned DB types exhibits specific features, mainly in terms of overall size and sequence redundancy, that might in turn considerably affect two of the main issues in proteome bioinformatics, namely false discovery rate (FDR) assessment and protein inference, respectively. FDR calculation applies a probabilistic method that inherently takes into account the effects of multiple testing, by estimating the proportion of peptide-spectrum matches (PSMs) that are incorrect among all significantly identified PSMs. Several computational approaches have been developed to estimate the FDR at both peptide and protein level, usually exploiting the well-established target-decoy approach [40], although alternative statistical modeling approaches have also been developed [41], [42]. Advantages and limitations of the FDR approach in terms of quality, accuracy, and resolution have been critically discussed elsewhere [43], [44], [45]. The FDR applies globally to a set of PSMs, but single PSMs can also be associated with a *q*-value, defined as the minimal FDR of any PSM set that includes the given PSM [44]. Even though FDR estimation can be quite accurate and reproducible when a limited search space is concerned (e.g., a protein DB from a single organism), its resolution may significantly deteriorate when the search space complexity increases, as it occurs in proteogenomic and metaproteomic experiments, with a consequent reduction in sensitivity [19], [45], [46]. FDR accuracy and sensitivity are expected to be strongly influenced by the protein DB used [46], but this aspect has not been fully elucidated so far regarding metaproteomic data.

The second bioinformatic concern, which may have a considerable impact on metaproteome analysis, is represented by the ‘protein inference problem’, that is, how to assemble a list of peptides into a (reliable) list of proteins [47], [48]. When analyzing a single organism’s proteome, ambiguities in peptide-to-protein assignment can be generally due to the presence of different splice variants or cleavage products. Unfortunately, this scenario is even more complicated when dealing with a metaproteome. In fact, many peptides (called *degenerate* peptides) can be shared among homologous proteins from different species, or even among recurring functional domains [19]. Under a DB perspective, a higher redundancy or homology in protein sequences corresponds to a higher degeneracy in peptide identification, and thus to harder issues in protein inference. Additionally, most of the widespread software suitable for protein/peptide identification usually display only a subset of all possible protein identifications; therefore, a tedious manual inspection for protein assignment is required in order to not over- or under-report important functional and taxonomic information [49].

A simple but quite robust strategy to infer taxonomic information from (DNA or protein) sequence data is the so-called lowest common ancestor (LCA) approach [50]. According to this algorithm, a sequence is assigned to a given species only if it does not match with any other species contained in the sequence DB; conversely, if the sequence is shared among several species contained in the DB, all belonging to the same genus, the sequence is unambiguously assigned only to the genus level. Generally speaking, widely conserved sequences are always assigned to high-order taxa. When analyzing metaproteomics data, the LCA approach is clearly to be preferred over retrieving the taxonomic information using ‘classical’ protein inference algorithms, as usually these systems select arbitrarily only one among the diverse taxonomic possibilities, with consequent loss of information [49]. The LCA algorithm can be theoretically applied either at the peptide or protein level: in the first case, LCA analysis should provide the most accurate results, in view of the peptide-centric nature of shotgun mass spectrometry (MS); in the second case, as discussed above, the previous application of a protein inference algorithm not specifically suited for metaproteomics might introduce significant biases. The forerunner of LCA software, MEGAN, was originally developed for metagenomic data, but it can also be extended to metaproteomics [51], [52], [53]. Usually, in a preprocessing step, protein/peptide sequences are compared against the NCBI database using BLAST, and MEGAN is then used to compute and explore the taxonomical content of the data set. A recent achievement of the metaproteomics research community is the Unipept web application, which supports biodiversity analysis of metaproteome samples using tryptic peptide information obtained from shotgun MS/MS experiments, by retrieving all occurrences of the given peptides in UniProtKB records; taxon-specificity of the tryptic peptide is successively derived from these occurrences using a novel LCA approach [54]. To date, a critical evaluation of Unipept and/or MEGAN for the taxonomic profiling of metaproteomic data has not yet appeared in the literature; furthermore, a possible influence of the DB choice on LCA results has not been investigated so far.

In this work, we aimed to assess the impact of different protein DBs on the metaproteomic investigation of a lab-assembled microbial mixture, composed by bacterial and eukaryotic species with various structural features and different levels of previous genomic characterization. In fact, the use of an ‘artificial microbiota’ enables to assess the reliability and completeness of metaproteomic data, thanks to the *a priori* knowledge of the exact species composition of the mixture. Total genomic extracts from the microbial mixture and from each of the cultured species were subjected to next generation sequencing (NGS) in order to obtain metagenomic- and genomic-derived DBs, which were interrogated along with publicly available DBs, parsed according to different taxonomy filters, to analyze shotgun metaproteomic data. The results obtained were evaluated by performing: i) a quantitative comparison of peptide identifications; ii) a critical assessment of FDR behavior and peptide degeneracy; iii) an in-depth analysis of taxonomic attribution reliability, carried out with MEGAN and Unipept software.

## Materials and Methods

### Microbial samples

Identity and features of the microbial strains used in this study are detailed in Table 1. *P. multocida* was kindly provided by Dr. Gavino Marogna (Istituto Zooprofilattico Sperimentale della Sardegna), *R. glutinis* by Prof. Ilaria Mannazzu (Department of Agricultural Sciences, University of Sassari), *L. casei*, *L. acidophilus, P. pentosaceus* and *S. cerevisiae* by Dr. Pasquale Catzeddu and Dr. Manuela Sanna (Porto Conte Ricerche), *B. laterosporus* by Dr. Luca Ruiu (Bioecopest Srl), whereas *E. faecalis* and *E. coli* were available in the laboratories of the Department of Biomedical Sciences, University of Sassari. When this study was performed, none of the specific microbial strains listed in Table 1 had its genome sequenced and deposited, except *B. laterosporus*. Microbial cultures were grown at 37°C to stationary phase using the appropriate standard medium for each microorganism, and colony-forming units (CFU) counting was used to estimate the amount of viable microbial cells. The microbial cultures were divided into aliquots (approximately 10^9^ CFU each), which were washed three times in PBS, pelleted, and stored at –80°C until used. A nine-organism microbial mixture (9MM) was then assembled by merging an aliquot of each microbial pellets according to the procedures described below.

**Table 1:** Microorganisms used in this study.

Species	Cell type	Source	Genome size
*Escherichia coli*	Gram-negative bacillus	Field isolate	4600 Kb
*Pasteurella multocida*	Gram-negative coccobacillus	Field isolate	2250 Kb
*Brevibacillus laterosporus*	Gram-variable bacillus	LMG 15441	5180 Kb
*Lactobacillus acidophilus*	Gram-positive bacillus	LMG 9433	1993 Kb
*Lactobacillus casei*	Gram-positive bacillus	LMG 6904	2900 Kb
*Enterococcus faecalis*	Gram-positive coccus	Field isolate	3218 Kb
*Pediococcus pentosaceus*	Gram-positive coccus	Field isolate	1832 Kb
*Rhodotorula glutinis*	Yeast	Field isolate	20300 Kb
*Saccharomyces cerevisiae*	Yeast	CBS 1171	12068 Kb

### DNA extraction and sequencing

DNA of single bacterial species was extracted according to a procedure hereafter called method A, based on detergent lysis and lysozyme treatment according to the DNeasy Blood & Tissue Kit protocol (Qiagen, Hilden, Germany), whereas yeast DNA was extracted according to a procedure hereafter called method B, comprising a strong detergent pretreatment combined with freeze-thawing and bead beating steps (as previously described by Harju and coworkers [55]) followed by the Gentra Puregene kit protocol (Qiagen). Furthermore, two identical replicates of the 9MM were assembled by merging 10^9^ CFU cell pellets from the nine microorganisms mentioned above. Then, the first 9MM replicate was subjected to extraction according to method A (9MM-A), while the second according to method B (9MM-B). The extracted DNA was quantified using the Nanodrop 2000 (Thermo Scientific, Waltham, MA, USA), and quality was assessed by agarose gel electrophoresis.

The 11 DNA extracts (9 individual microbes, 9MM-A and 9MM-B) were then subjected to NGS. Libraries were generated using the Illumina® TruSeq™ DNA Sample Preparation Kit (San Diego, CA, USA) according to the manufacturer’s protocol with minor modifications. Briefly, genomic DNA was fragmented in an ultrasonic bath (Elmasonic S, Elma, Singen, Germany). After ligation to the adapters and gel purification of DNA ranging between 300 and 400 bps, the libraries were subjected to 15-20 PCR cycles to enrich the DNA fragments with adapters ligated to both ends. The PCR products were purified and evaluated using the High Sensitivity DNA chip on an Agilent Technologies 2100 Bioanalyzer (Santa Clara, CA, USA). Normalized sample libraries were pooled and subjected to hybridization and cluster generation step on a v1 flow cell using the cBOT cluster generation station, according to the Illumina TruSeq PairedEnd Cluster Kit protocol. Libraries were sequenced (six samples per lane) with an expected coverage of at least 40X for each single microorganism except for *R. glutinis* (about 12X). The 9MM extracts were sequenced with a higher coverage (only two samples per lane) to achieve a better sequencing depth. DNA sequencing was performed with the Illumina HiScanSQ sequencer, using the paired-end method and 76 runs of sequencing. The 9MM-A and 9MM-B metagenome sequences have been deposited in the NCBI BioSample repository (http://www.ncbi.nlm.nih.gov/biosample), with the accession numbers 2352454 and 2352511, respectively.

### Genome/metagenome gene finding, annotation and six-frame translation

Reads were assembled *de novo* into scaffolds using Velvet 1.2 [56], choosing the best K-mer values for each assembly to obtain nine genome drafts and two metagenome drafts. As detailed in Data S1, all the *de novo* drafts of the single microorganisms showed a N50 length >30 kbp and a coverage higher than 39X, except for *R. glutinis* (4910 bp and 12.6X, respectively, probably due to its wider genome); 9MM-A metagenome reads showed an assembly quality equivalent to the single genome sequences, whereas N50 length of the 9MM-B draft was significantly lower (< 1000). The putative coding sequences (CDS) were identified with Prodigal 2.60 [57]. Each CDS was annotated evaluating the homology by BLAST search against TrEMBL Protein Database Release 2012_10 (E-value ≤ 10^−8^) [58]. Moreover, each genome draft was translated in all six frames using the perl script translateWholeGenomeMultiChromosome.pl available at http://proteomics.ucsd.edu/Downloads/.

### Protein extraction and shotgun MS analysis

The 9MM was assembled as follows: the first microbial pellet was resuspended in 500 µl of pre-heated (95°C) extraction buffer (2% SDS, 20 mM Tris-HCl pH 8.8); after careful pipetting, the microbial suspension was then added to a second microbial pellet, and the procedure was sequentially repeated until the ninth pellet was resuspended and mixed. The 9MM was incubated at 95°C for 20 min in agitation (500 rpm) in a Thermomixer. Next, a stainless steel bead (5 mm diameter, Qiagen, Hilden, Germany) was added. The sample was then subjected to sequential incubations (5 min at –80°C, 5 min at 95°C, 1 h at –80°C) followed by bead beating (10 min at 30 cycles/s in a TissueLyser mechanical homogenizer, Qiagen), further incubations (5 min at –80°C, 5 min at 95°C), and a final bead beating step (3 cycles of 3 min each at 30 cycles/s in the TissueLyser). The sample was centrifuged at 14000 rpm for 10 min at 4°C and the whole supernatant was collected. Protein quantification was carried out by means of the 2-D Quant Kit (GE Healthcare, Little Chalfont, UK).

According to previous studies [59], [60], [61], [62], two complementary approaches were chosen for sample preparation prior to MS, namely filter-aided sample preparation (FASP) [63] and protein precipitation followed by in-solution digestion (PPID). Two aliquots of the 9MM protein extract were therefore processed in parallel according to these two procedures, as described previously [62].

The two derivative peptide mixtures were then analyzed by LC-MS/MS using an LTQ-Orbitrap Velos (Thermo Scientific) interfaced with an UltiMate 3000 RSLCnano LC system (Dionex, Sunnyvale, CA, USA, now part of Thermo Scientific), according to a previously described method [64]. Briefly, four micrograms of each peptide mixture were separated at 35 °C using a 75-µm ID × 25 cm C18 column (Acclaim PepMap RSLC C18, 75 µm15 cm nanoViper, 2 µm, 100 Å, Dionex) at a flow rate of 300 nL/min, using a 280 min gradient from 1 to 50% eluent B in eluent A, where B is 0.2% formic acid in 95% acetonitrile, and A is 0.2% formic acid in 5% acetonitrile. Full-scans were performed in the Orbitrap with resolution of 30,000 at 400 m/z, and the 10 most intense ions of every scan were selected and fragmented. Higher Energy Collisional Dissociation (HCD), performed at the far side of the C-trap, was used as fragmentation method by applying a 40% value for normalized collision energy, an isolation width of m/z 3.0, a Q-value of 0.25, and an activation time of 0.1 ms. Finally, the two Thermo raw files obtained from the two peptide mixtures were merged in order to maximize protein sequence coverage and thus metaproteome analysis depth. Individual peptide/PSM identification values from FASP and PPID analyses are shown in Data S2. Technical reproducibility among runs, measured by performing an additional experiment (data not shown) and calculating the R-squared value by plotting the number of PSMs for a given protein in run 1 against the number of PSMs for the same protein in run 2, was 0.9954.

### Protein database construction and metaproteome bioinformatics

Thirteen protein DBs were used for protein/peptide identification from MS data, as described in Table 2.

**Table 2:** Database used for peptide identification from MS spectra.

Database acronym	Original database	Version/ update	Taxonomy/ Processing	Number of sequences	Average computing time per run (min)
**NCBI-BFV**	NCBI	Dec 2012	Bacteria, Fungi, Viruses	16,175,389	817
**TrEMBL-BFV**	UniProtKB/ TrEMBL	2012_10	Bacteria, Fungi, Viruses	21,602,141	1002
**SP-BFV**	UniProtKB/ Swiss-Prot	2012_11	Bacteria, Fungi, Viruses	375,700	28
**NCBI-G**	NCBI	Dec 2012	8 selected genera	895,743	213
**NCBI-S**	NCBI	Dec 2012	9 selected species	554,718	219
**TrEMBL-G**	UniProtKB/ TrEMBL	2012_10	8 selected genera	2,622,251	269
**TrEMBL-S**	UniProtKB/ TrEMBL	2012_10	9 selected species	2,198,849	247
**SP-G**	UniProtKB/ Swiss-Prot	2012_11	8 selected genera	37,708	9
**SP-S**	UniProtKB/ Swiss-Prot	2012_11	9 selected species	33,130	8
**Meta-PA**	Matched metagenome		CDS prediction + TrEMBL annotation	24,673	10
**Meta-6FT**	Matched metagenome		six-frame translation	90,306	17
**SGA-PA**	Single genomes assembly		CDS prediction + TrEMBL annotation	52,455	10
**SGA-6FT**	Single genomes assembly		six-frame translation	54,948	28

The first nine DBs were assembled starting from publicly available sequences derived from NCBI, UniProtKB/SwissProt (hereafter simply called SwissProt), and UniProtKB/TrEMBL (hereafter simply called TrEMBL) records, using the Database Manager tool included in Mascot Server (version 2.4, Matrix Science, London, UK), and applying one of the three following taxonomy filters: Bacteria, Fungi, Viruses (BFV, corresponding to NCBI taxonomy IDs 2, 4751, and 10239), selected genera (*Brevibacillus, Escherichia, Enterococcus, Lactobacillus, Pasteurella, Pediococcus, Rhodotorula,* and *Saccharomyces*, corresponding to NCBI taxonomy IDs 55080, 561, 1350, 1578, 745, 1253, 5533, and 4930), or selected species (*B. laterosporus, E. coli, E. faecalis, L. acidophilus, L. casei group, P. multocida, P. pentosaceus, R. glutinis, S. cerevisiae*, corresponding to NCBI taxonomy IDs 1465, 562, 1351, 1579, 655183, 747, 1255, 5535, 4932). The taxonomy *L. casei group* was preferred to *L. casei* (species) due to the very high level of sequence similarity and some ambiguity in taxonomic boundaries within the species comprised in this taxonomic group.

The remaining four DBs were constructed from genomic and metagenomic data experimentally obtained in this study. Specifically, the single predicted and annotated (PA) genomes assembly DB (SGA-PA) was obtained by concatenating in a single FASTA file the protein sequences obtained from each individual microbe upon CDS prediction and TrEMBL annotation, while the PA metagenome DB (Meta-PA) was obtained by concatenating in a single FASTA file the protein sequences obtained upon NGS of the two 9MM extracts, CDS prediction and TrEMBL annotation. Finally, the genome drafts of the nine sequenced microbes and the 9MM metagenome draft were also processed in an alternative way based on *naïve* six-frame translation, thus generating SGA-6FT and Meta-6FT DBs, respectively. As expected, the number of amino acid residues of the 6FT DBs was almost six time bigger than that of the corresponding PA DBs (specifically, 4.2 million residues for Meta-PA versus 26.1 for Meta-6FT, and 12.9 million residues for SGA-PA versus 84.2 for SGA-6FT). Features and composition of the in-house Meta-PA and SGA-PA DBs were as follows. The percentage of annotated proteins were 71% and 54% of the overall protein sequences, and the number of non-redundant protein sequences within each DB amounted to 13270 and 27164 for Meta-PA and SGA-PA, respectively. Among these, 96% and 91% were correctly attributed to the species actually present in the 9MM, respectively. Concerning the species distribution of the protein sequences contained into the two DBs according to TrEMBL annotations (Data S3), in the Meta-PA DB over 90% of protein sequences were from only 4 species (*B. laterosporus*, *P.multocida*, *L. casei group*, and *L. acidophilus*, representing 36%, 27%, 22% and 9% of the total, respectively), with a significant depletion in yeast sequences (e.g. only 1 from *S. cerevisiae*), whereas in the SGA-PA DB the abundance of the 9 actually present species ranged from 3 to 21% of the overall protein sequences.

Finally, a DB containing common contaminants (available at http://maxquant.org/contaminants.zip) was also used as a control for environmental and trypsin contamination.

The Proteome Discoverer platform (version 1.3.0.339, Thermo Scientific), interfaced with an in-house Mascot server, was used for data parsing and protein identification, according to the following criteria: Enzyme Trypsin, Maximum Missed Cleavage Sites 2, Precursor Mass Tolerance 10 ppm, Fragment Mass Tolerance 0.2 Da, Cysteine Carbamidomethylation as Static modification, N-terminal Glutamine conversion to Pyro-glutammic Acid, Methionine Oxidation and N-terminal Acetylation as Dynamic Modifications. The Percolator algorithm was used to calculate a *q*-value for each peptide/PSM, and then an FDR threshold was set at peptide level (generally <1%, see Results for details) based on Percolator *q*-value, according to Proteome Discoverer’s peptide confidence filtering. Peptides with rank >1 were not considered for analysis. Peptide and protein grouping according to Proteome Discoverer’s algorithms were allowed, applying strict maximum parsimony principle.

Peptide sequences were imported on Unipept (http://unipept.ugent.be/) [54], in order to infer taxonomic information about the identified peptides, and subjected to multi-peptide analysis setting the following parameters: “Equate I and L” and “Filter duplicate peptides”. Peptide sequences were also subjected to standard protein BLAST search against the NCBI-nr DB using blastp (http://blast.ncbi.nlm.nih.gov) with default parameters (included the automatic adjustment for short input sequences). BLAST output files (in xml format) were uploaded in MEGAN (MEtaGenome ANalyzer, version 4.70.4) to perform taxonomic analysis [52]. MEGAN parameters were left as default, except “Min support” which was set as needed (see Results for details). Data elaboration was carried out using Microsoft Excel (Redmond, WA, USA). Venn diagrams were designed by means of Venny (http://bioinfogp.cnb.csic.es/tools/venny/index.html) or Venn Diagram Plotter (http://omics.pnl.gov/software/VennDiagramPlotter.php). MS data, protein/peptide identifications list and other supplementary material are available in the Peptide Atlas repository at http://www.peptideatlas.org/PASS/PASS00194.

## Results

### Global experimental design

**Figure 1:**
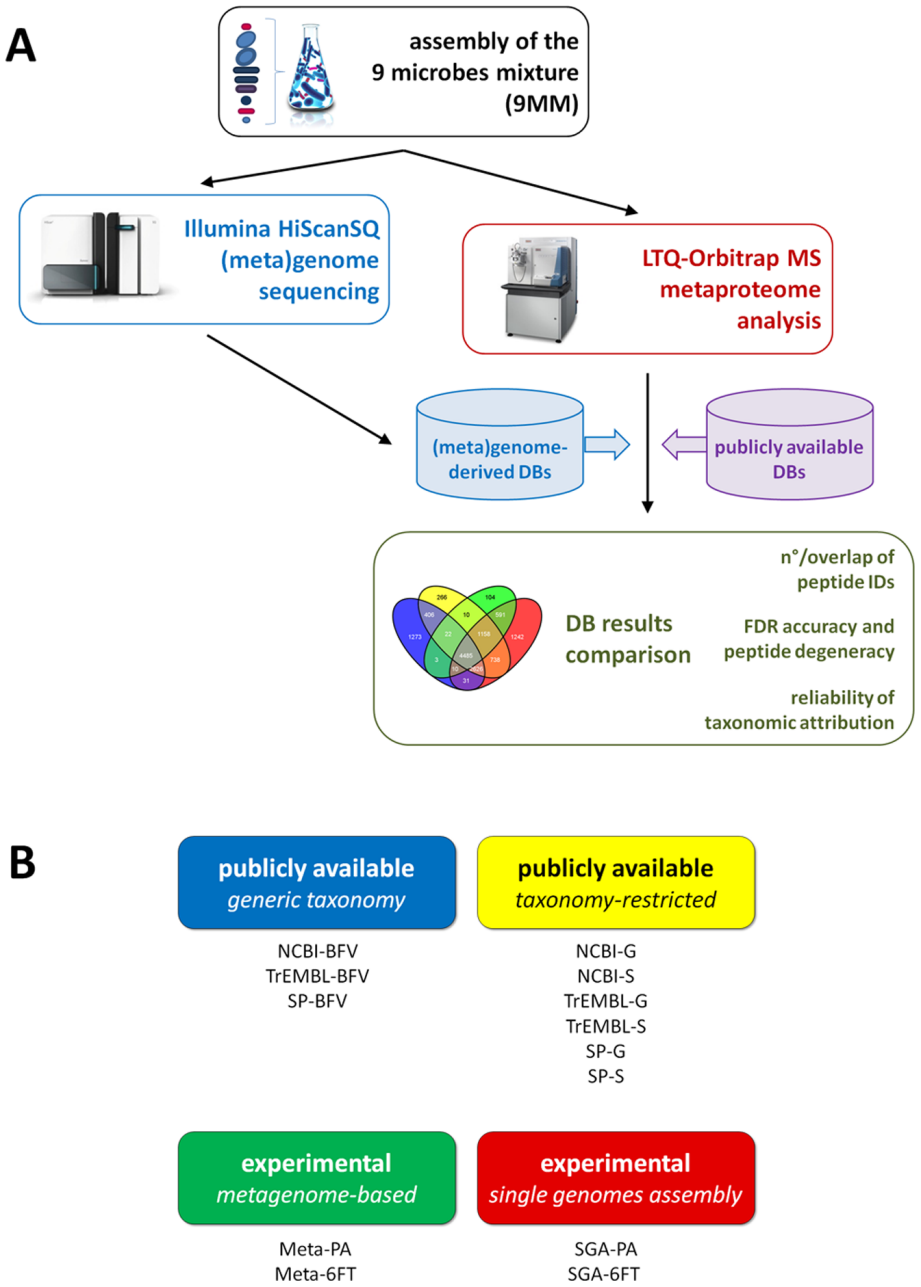
Schematic illustration of the study workflow. A) Experimental design. B) Database classes examined.

The study was designed as schematized in Figure 1A. As a first step, a nine strains microbial mixture (9MM) was assembled, including seven prokaryotes and two eukaryotes with heterogeneous structural features, as summarized in Table 1. In order to simulate the variability in the level of sequence information that might be encountered in environmental microbiomes, the selected microorganisms were either whole-genome sequenced reference strains, unsequenced field isolates, or belonging to species lacking a previous genomic characterization (i.e., *R. glutinis*). As a second step, genomes extracted from the 9 individual strains and from the 9MM were subjected to Illumina NGS, in order to generate genome- and metagenome-derived protein DBs (see Materials and Methods for details). As a third step, the 9MM metaproteome was analyzed by shotgun LTQ-Orbitrap MS, and MS data were searched against publicly available and matched experimental DBs. As a fourth step, the information achieved interrogating a total of 13 different DBs was comparatively evaluated in respect to: number and overlap of peptide identifications; FDR behavior and peptide degeneracy; and reliability of taxonomic attribution (using MEGAN and Unipept software).

Specifically, four main DB classes were considered for comparison, each one corresponding to a different experimental approach that might be used in a metaproteomics study (as depicted in Figure 1B): i) public DBs (namely, NCBI, SwissProt and TrEMBL) with generic taxonomic indications (all microbial sequences, i.e. those belonging to *Bacteria*, *Fungi*, and *Viruses*, abbreviated as BFV), an approach needed when no precise taxonomic information and/or matched genome sequencing data are available for the microbiome under study; ii) protein sequences selected from the above mentioned public DBs, based on taxonomic information (referred to as ‘taxonomy-restricted’ DBs, parsed at genus, G, or species, S, level) which may derive from previous 16S rRNA gene sequencing or metaproteomic information; iii) matched metagenome sequence DBs (named ‘Meta’ DBs), experimentally obtained from whole metagenome sequencing of the same microbiome subjected to metaproteomic analysis; iv) assembly of experimentally obtained individual genome sequences from the main species included in the microbiome (named ‘single genomes assembly’, SGA), an approach that requires isolation of each strain of the culturable microbiome. A further distinction must be made concerning genome data processing: both the metagenome and the single genomes were subjected either to coding sequence prediction and annotation (PA) or to *naïve* six-frame translation (6FT), thus generating four different experimental DBs.

### Comparison of metaproteomic data obtained using different protein databases

**Figure 2:**
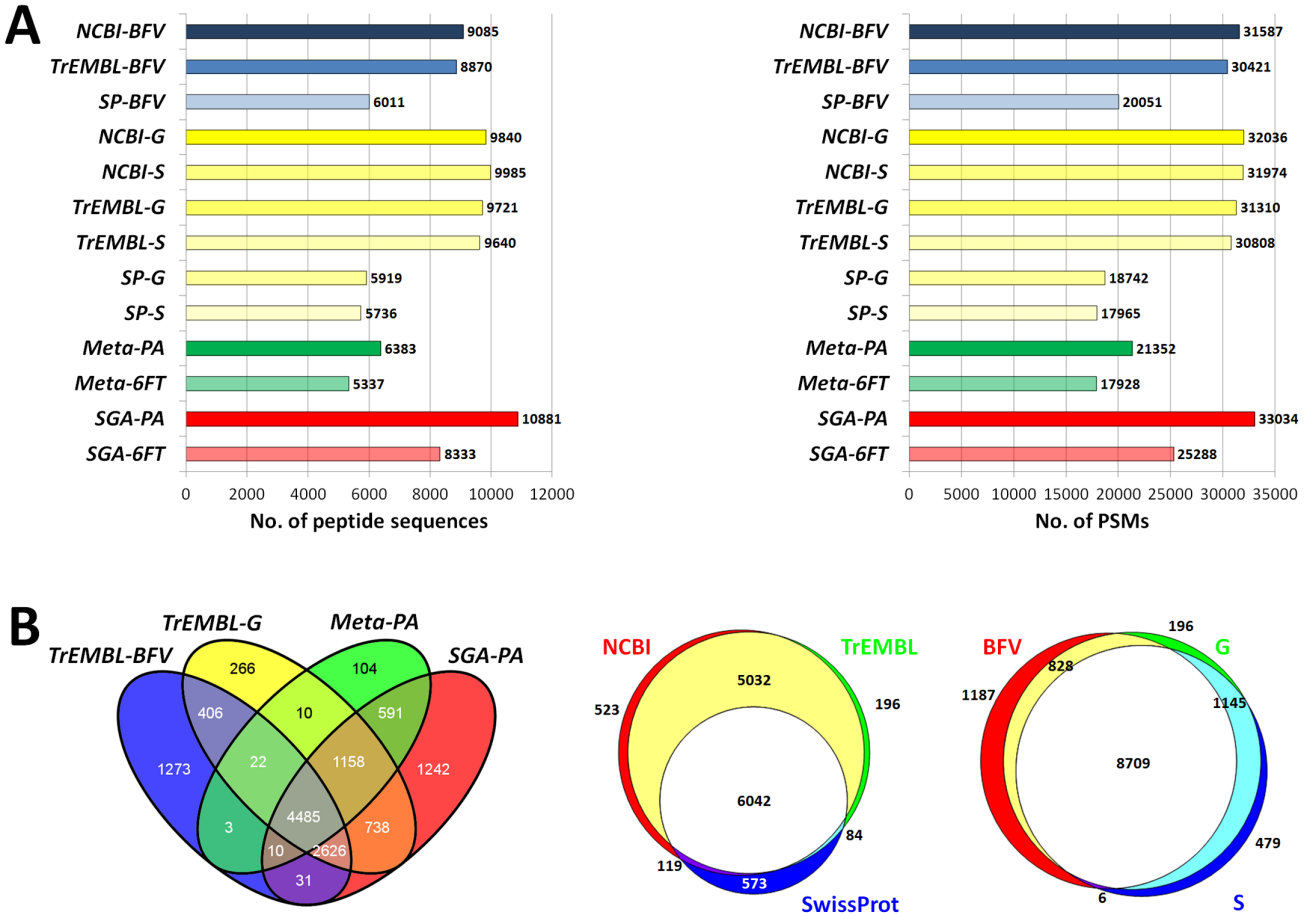
Comparison of metaproteomic data obtained with different databases. A) Number of peptide sequences (left) and peptide-spectrum matches (PSMs, right) identified in the 9MM using different sequence databases (FDR<1%). B) Left, Venn diagram illustrating the peptide distribution among four different DB classes. Center, Venn diagram illustrating the peptide distribution among all NCBI-, TrEMBL- and SwissProt-based DBs used in this study. Right, Venn diagram illustrating the peptide distribution among all DBs with generic microbial taxonomy (BFV), genus-specific taxonomy (G), and species-specific taxonomy (S).

Figure 2A illustrates the comparison among the peptide identification data achieved by searching the MS spectra against the 13 DBs described above, using FDR<1% as a threshold. The use of SGA-PA led to the identification of the higher number of peptides (left), while SwissProt-based DBs provided the least satisfactory results. Similar results were obtained according to the number of peptide-spectrum matches (PSMs; right). The amount of peptide identifications achieved with the metagenome-derived DB was slightly higher than with SwissProt, but clearly lower than with public non-manually annotated NCBI and TrEMBL DBs. Furthermore, ‘taxonomy-restricted’ DBs from NCBI and TrEMBL performed better than the corresponding DBs with wider taxonomy. It should also be noted that, as indicated in Table 1, the average computing time needed for the DB search differed dramatically among the DBs, proportionally to each DB size. On the whole, 12911 different peptide sequences were identified by searching MS spectra against all DBs described above.

Four DBs were then selected as representative of the four main DB classes described above. Specifically, two were TrEMBL-based DBs, and two were (meta)genome-based DBs annotated against TrEMBL. The intersections among the peptide sequences identified with each DB were calculated and illustrated by means of a Venn diagram (Figure 2B, left). Surprisingly, only about one-third of the identified peptide sequences were common to all DBs, while 22% were unique to a single DB (of which nearly 90% were unique to TrEMBL-BFV or SGA-PA). Meta-PA identifications were common to SGA-PA at 98%, whereas the specific increment obtained with Meta-PA compared to the public DBs (given by the peptide sequences found only using Meta-PA and not detected using any publicly-available DB) could be estimated at 6%. Furthermore, 68% of peptide sequences were in common between TrEMBL-BFV and TrEMBL-G.

When comparing DBs according to the public DB of origin (Figure 2B, center), approximately half of the peptides were common to NCBI, TrEMBL and SwissProt; NCBI and TrEMBL shared over 90% of the identified peptides, while about 8% of SwissProt peptide sequences (5% of the total) were not identified in the other DBs. As far as different taxonomy filters are concerned (Figure 2B, right), 70% of peptide identifications were common to all DBs, but the use of genus/species-specific DBs led to a 17% increase in identifications compared to search against a general microbial taxonomy (BFV).

The performances of 6FT DBs were also evaluated. A total of 5337 peptides were identified by searching MS spectra against Meta-6FT, of which 117 (2%) were unique when compared to the corresponding annotated DB (Meta-PA); SGA-6FT allowed the detection of 8333 peptides, of which 757 (9%) had not been found using SGA-PA. On the whole, the employment of 6FT DBs enabled 783 additional identifications (6% increase).

### Evaluation of FDR behavior and peptide degeneracy across different databases

**Figure 3:**
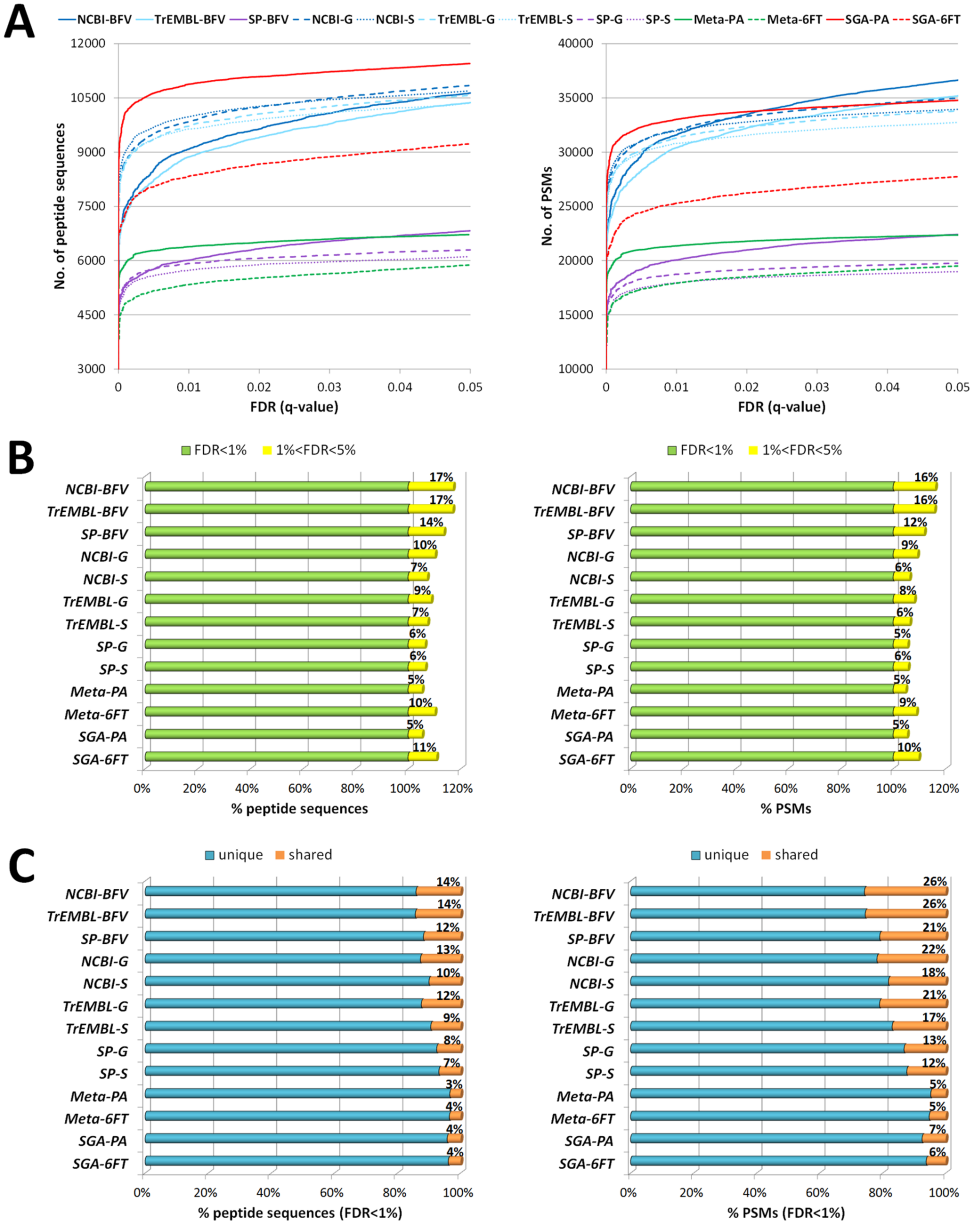
Evaluation of FDR behavior and peptide degeneracy using different databases. A) Diagram plotting the number of peptides (left) and PSMs (right) identified with each database as a function of FDR thresholds based on the Percolator *q*-values. B) Bar graph showing the percentage increment in peptide (left) and PSM (right) identifications achieved with each database when increasing the FDR threshold from 1 to 5%. C) Bar graph illustrating the percentage of shared peptides (left) and PSMs (right) identified with each database at FDR<1%.

Another aim of this study was to investigate how FDR behavior and peptide degeneracy are influenced by the particular DB used for metaproteome analysis. To evaluate FDR behavior, the number of peptides (Figure 3A, left) and PSMs (right) identified with each DB were plotted as a function of FDR thresholds based on the Percolator *q*-values, as previously described by Spivak and colleagues [65]. As a result, DBs could be distinguished into two groups based on the typical trend of their *q*-value curves: the first comprising all publicly available DBs with generic taxonomy (NCBI-BFV, TrEMBL-BFV and SP-BFV), whose curve kept on rising much longer compared to the remaining DBs, that tended considerably more rapidly to a *plateau*. Interestingly, the FDR evolution was quite different if either peptide sequences or PSMs were considered. For instance, SGA-PA achieved the higher number of peptide identified at any FDR, whereas in terms of PSMs the same DB passed from giving the best results at 1% FDR to being only the fourth best DB at 5% FDR. The increment in peptide/PSM identifications when increasing the FDR threshold from 1 to 5% was also evaluated (Figure 3B), and it was observed that the public DBs with generic taxonomy consistently yielded the highest percentage of additional hits when increasing the FDR threshold. Another significant observation could be made concerning 6FT DBs, which showed a two-fold percentage increase compared to the corresponding PA DBs when the FDR threshold was raised to 5%.

Furthermore, the degree of peptide degeneracy related to each DB was estimated by calculating the percentage of shared (or degenerate) peptides/PSMs. According to Proteome Discoverer’s algorithms, after protein identities are deduced from a set of identified peptides, proteins are grouped according to the peptide sequences identified for the proteins (in this case allowing the “Strict Maximum Parsimony Principle” option), and a master protein is reported for each protein group, which has been identified by a set of peptides that are not included (all together) in any other protein group. Each identified peptide can be therefore matched either with a single protein group (called ‘unique peptide’) or with multiple protein groups (called ‘shared peptide’). In this context, the percentage of shared peptides out of the overall identifications gives an indication of the degeneracy associated to a particular DB. As shown in Figure 3C, in general the percentage of shared PSMs (right) was higher when compared to the percentage of shared peptides (left) measured for the same DB (with FDR<1%). Moreover, experimental DBs exhibited significantly lower percentages of shared peptides (and even lower for PSMs) when compared to publicly available DBs. Among the latter, the peptide degeneracy decreased, as expected, according to the following order: NCBI>TrEMBL>SwissProt and BFV>G>S.

### Reliability of taxonomic attribution by Unipept and MEGAN analysis of metaproteomic data

The metaproteomic data generated in this work were then used to evaluate the reliability of the taxonomic attribution of peptide identifications, with the aim of assessing the influence exerted by the DB choice in this type of investigations. Such evaluation was possible due to the *a priori* knowledge of the taxonomic composition of the lab-assembled 9MM. Specifically, the peptide sequences identified using the different DBs were parsed by means of two software enabling taxonomic analysis according to the LCA approach, namely Unipept [54] and MEGAN [52]. It is worth noting that MEGAN requires a preliminary BLAST search of the identified peptide (or protein) sequences, since a BLAST file is needed as the input. Furthermore, MEGAN “Min Support” filter (that is, the number of reads/peptides that must be assigned to a taxon so that it appears in the results) was initially set to 1, according to Rudney *et al.* [53].

**Figure 4:**
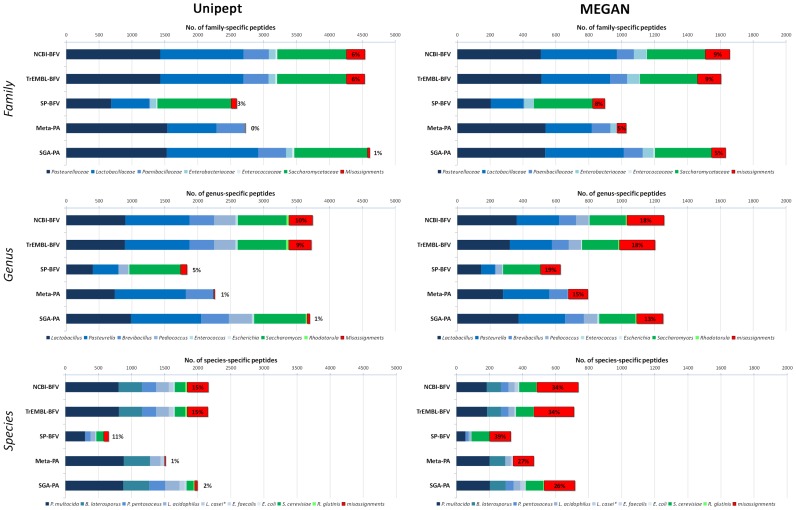
Reliability of taxonomic attribution using Unipept and MEGAN. Bar graphs showing taxonomic distribution of family (top), genus (middle) and species (bottom) specific peptides identified with different DBs, according to Unipept (left) or MEGAN (right) LCA analysis. Red rectangles illustrate misassignments (i.e. attributions to taxa not actually present in the 9MM), with indication of their percentage for each DB. Bacterial taxa are represented by shades of blue, whereas yeast taxa by shades of green.

Figure 4 comparatively illustrates the number of peptides detected as specific to family (top), genus (middle) or species (bottom) level upon Unipept (left) or MEGAN (right) analysis, identified with five different DBs. Peptide distribution among the prokaryotic (blue) and eukaryotic (green) strains included in the 9MM was also taken into account, as well as the incorrect attributions (denominated ‘misassignments’, in red). Further details are available in Data S4-S5. Genus/species-specific DBs were excluded from this comparison because it would have been superfluous to assess taxonomy attribution reliability when a specific “taxonomy filter” had been already set *a priori*, and therefore the number of misassignments had been “forced” to be zero. In general, the number of taxon-specific identifications decreased proportionally to the degree of taxonomic detail (for instance, nearly 4500, 3500, and 2000 peptides could be found with family, genus, and species specificity with NCBI-BFV, respectively). Moreover, a higher amount of taxon-specific peptides could be yielded with Unipept analysis compared to MEGAN (e.g. up to over 4500 family-specific peptides with Unipept versus less than 1800 with MEGAN). The impact of taxonomic ‘misassignments’ was also evaluated. As a result, Unipept demonstrated a higher reliability, since the average percentage of incorrect attributions was 3%, 5% and 9% (at the family, genus and species level) compared to respective percentages of 7%, 17% and 32% with MEGAN. Among DBs, Meta-PA provided the most specific results, due to the lowest rate of misassignments, whereas NCBI-BFV and TrEMBL-BFV performed worse in this respect. With regard to the distribution of the taxon-specific peptides among the different microbial strains, no yeast-specific peptides could be identified using Meta-PA, because of the total lack of eukaryotic sequences in this DB. Bacterial family distribution was instead comparable among all DBs. Going down to the species level, the best coverage was achieved by SGA-PA, followed by NCBI-BFV and TrEMBL-BFV which provided similar results. Conversely, SP-BFV failed to detect peptides belonging to the species with lower level of genomic characterization (as *B. laterosporus* and *R. glutinis*, since no protein sequences from these species were included within SwissProt records at the time of this study). *E. faecalis* and *E. coli* were significantly underrepresented with all DBs.

**Figure 5:**
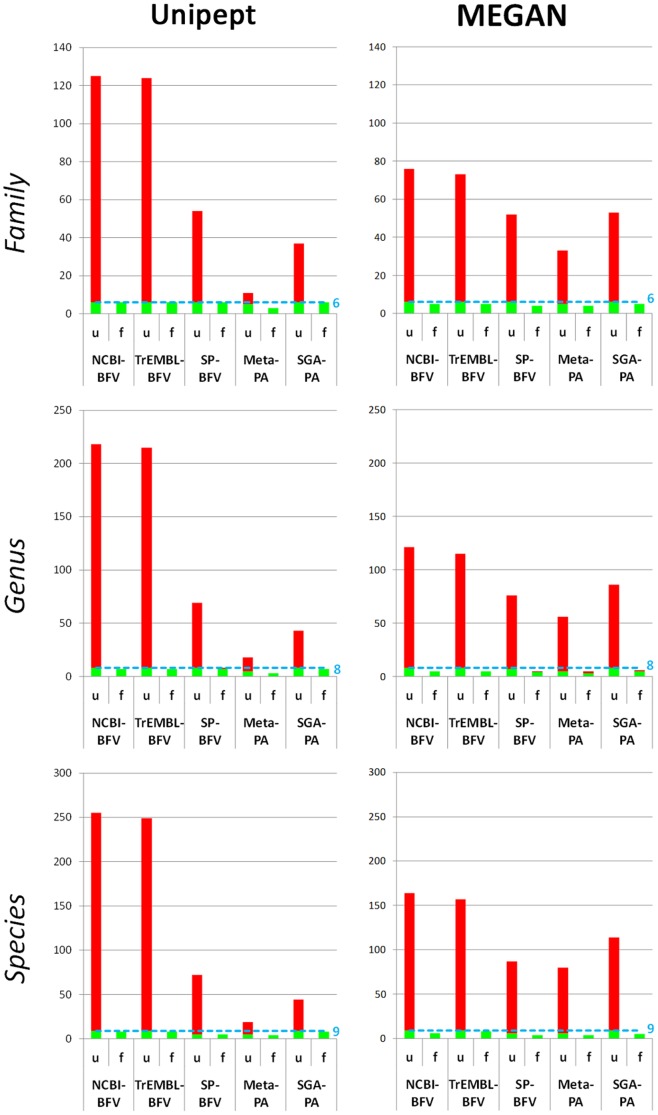
Improvement of the reliability of taxonomic attribution upon data filtering. Histograms showing the number of families (top), genera (middle) and species (bottom) detected upon Unipept (left) or MEGAN (right) LCA analysis using different DBs, before and after the application of a filter based on the number of taxon-specific peptides (u, unfiltered; f, filtered). The threshold was set to 0.5% of the overall number of peptides unambiguously assigned to a taxon at a particular taxonomic rank level (family, genus or species). Correct and incorrect attributions are represented in green and red, respectively. The light blue lines and numbers correspond to the number of families, genera or species actually present in the 9MM.

In addition, when considering the overall number of families, genera, and species found with the different DBs, results were very far from the expected value. As an example, Unipept analysis of peptides identified using TrEMBL-BFV revealed the (purported) presence of 124 different families, 215 different genera, and 249 different species within the 9MM (as detailed in Data S4; in this case, MEGAN did generally provide a lower number of false positives when compared to Unipept). This, together with the non-negligible percentage of misassignments described above, demonstrates that taxonomic information gathered without adequate filtering can provide confounding results, dramatically decreasing the reliability of metaproteomic data. In keeping with this, an empirical filter was devised with the aim of eliminating false positive attributions and making the final result as similar as possible to the actual 9MM composition. Upon iterative analyses, a threshold corresponding to 0.5% of the total number of taxon-specific peptides was set, thus defining the taxa exhibiting a number of peptides below such value as false positives. As shown in Figure 5 (‘u’ indicates unfiltered data, whereas ‘f’ indicates filtered data), in most cases the application of this filter allowed the elimination of all incorrect taxa (in red) without (or with only slight) loss of information about the actually present strains (in green). Distribution of taxon-specific peptides and misassignments after filtering is illustrated in detail in Data S5.

We also sought to investigate the taxonomic features of 6FT-unique peptide sequences. In fact, 783 peptides were identified only using 6FT DBs (Meta-6FT or SGA-6FT), since their sequence was absent from the corresponding predicted and annotated DBs. To this aim, the 6FT-unique sequences were classified based on the individual genome of origin (this information was available only for SGA-6FT), as well as subjected to BLAST sequence similarity analysis (both Meta-6FT and SGA-6FT). As a result, among 675 6FT-specific peptides detected using SGA-6FT, 77% matched with sequences belonging to *R. glutinis* genome, followed by 12% from *S. cerevisiae* (thus nearly 90% were from yeast sequences), about 4% each from *P. multocida* and *L. casei*, and an additional 4% from the remaining microbes. This result was confirmed by BLAST analysis, since 72% of the aligned sequences were found as significantly homologous to yeast sequences (21 to *R. glutinis*, 10 to *S. cerevisiae* and 34 to other *Fungi*).

## Discussion and Conclusions

A formidable effort is currently being made to develop bioinformatic strategies able to tackle issues in metaproteomic data analysis [19], [20], [30], [31], [66]. The results presented here further highlight that the use of large and complex DBs required for multispecies samples (such as microbial communities) poses significant challenges in the implementation and optimization of search-decoy approaches for FDR calculation. Our data also suggest that peptide/PSM identification significance thresholds are strongly influenced by DB size and redundancy, even when a post-search algorithm using semi-supervised machine learning (such as Percolator) is used. In fact, the use of ‘taxonomy-restricted’ DBs led to a higher number of peptide identifications when compared to those obtained with the same DBs with wider taxonomy (and thus larger size). This may seem quite surprising, given that ‘taxonomy-restricted’ DBs were just a subset of the corresponding ‘general’ DBs, containing no additional sequences when compared to the latter. Specifically, most of the peptide sequences uniquely detected with ‘taxonomy-restricted’ DBs were not identified using the corresponding ‘general’ DB, since these were discarded being below the 1% FDR threshold. Also the poorer performance of 6FT DBs when compared to the corresponding PA DBs may be explained in a similar way, since the former are almost six time bigger than the latter. In this respect, the use of alternative search-decoy strategies as those described by Blakeley et al. [46] and, even more recently, by Jagtap et al. [66] might partially address this problem and lead to an increase in peptide identifications, and may be the target of future studies. The same phenomenon could be observed for SwissProt when compared to TrEMBL (Figure 2B): TrEMBL provided a much higher absolute number of identifications (mostly due to the lack of less characterized species within SwissProt), but the parallel use of SwissProt gave additional, unique information. Manually curated DBs offer also further advantages, including a higher level and quality of annotation concerning protein functions, processes, and localizations, which can be extremely useful in the functional perspective allowed by metaproteomics. It should also be noted that the results presented here were obtained using Percolator’s and Proteome Discoverer’s algorithms for FDR calculation and protein grouping, respectively. Several alternative, more sophisticated approaches are available to perform these post-processing operations [48], [67], [68], [69], [70] (and metaproteomics-targeted software will be hopefully developed in the near future), which might deliver significantly different data. Furthermore, the complexity of the lab-assembled microbial mixture used in this study was far from that of a typical “real-world” microbiome. This suggests that caution is required before extending the conclusions described here to the most heterogeneous environmental samples, and that further validation studies are needed to define an optimized pipeline for metaproteomic data analysis.

The information needed for generating a ‘taxonomy-restricted’ DB can be easily gathered by 16S-18S characterization, but a metaproteomic iterative approach can be also proposed, comprising a first search using a generic DB, sequentially followed by the identification of the main taxa of the microbiome of interest from metaproteomic data (using proper filters to improve reliability, as described in this work and discussed below), the construction of a customized, smaller DB, and a second search with this latter DB to improve metaproteome coverage. This iterative metaproteomic strategy (which differs from the ‘two-step method’ proposed by Jagtap and coworkers [66] in that the former is taxonomy-based) might be therefore successfully implemented without the need for additional genomic or metagenomic surveys.

Another issue that we chose to address is the critical comparison of the two main software, available to date, suitable for the taxonomic classification of metaproteomic data, namely Unipept and MEGAN. To the best of our knowledge, the data presented here represent the first comparative evaluation of tools enabling biodiversity analysis of metaproteome samples. For this purpose, Unipept appeared to be more straightforward (in terms of user-friendliness, speed of analysis, and output reliability). On the other hand, MEGAN can additionally provide functional and pathway information which are key for metaproteomic studies (but beyond the scope of this work). More specifically, two parallel MEGAN analyses were carried out (as suggested by MEGAN developers [51]): the first using peptide sequences as BLASTP input, and the second using the inferred protein sequences to avoid issues due to the extreme shortness of peptide sequences. The second analysis produced a higher amount of information, but reliability of taxonomic attributions was rather poor (Data S6), consistently to the protein inference issues that have to be expected in a metaproteomic experiment; therefore, we chose to use only the data obtained using peptide sequences for comparison with Unipept data, also taking into account the peptide-centric nature of shotgun proteomics. It has to be mentioned that modifying the MEGAN parameter “Min Score” (which was not changed from the default settings in this study) may have led to different results, especially when dealing with peptide sequences. In addition, an empirical threshold was established to filter taxonomic classification, in order to discard false positive attributions. In our case, this has been possible by analyzing a simple microbial community of known composition, and then searching for an optimized filter allowing the maximization of the real positive attributions and the minimization of the false positive ones. Specifically, the current version of Unipept does not allow the user to set a threshold (it should be done manually by parsing the csv output file); conversely, MEGAN includes a “Min Support” filter that can be easily modified according to the user’s need. In particular, only two interesting reports (from the same research group) described the use of MEGAN for metaproteomic data analysis [53], [71], and the first clearly stated that “because the number of reads in this proteomic dataset was considerably smaller than the thousands usual in a metagenomic dataset, the number of reads required for a taxon assignment was set to one”. Here, we demonstrate that using such a low threshold can give rise to a significant percentage of misassignments. Clearly, the particular threshold adopted in this study might not be adequate for more complex environmental samples; however, our results underline that the raw taxonomic data may contain a significant share of false positives, and therefore strongly suggest a critical examination of the results. These incorrect species attributions might be generally due to the incompleteness of the genomic characterization of the species contained in a given microbial community. For instance, several strains of species “A” have been sequenced, and therefore different sequence variants are available in a DB. Conversely, species “B”, related to species “A”, has been less studied, and a single strain has been sequenced. As a consequence, an unknown sequence polymorphism (or even an inaccuracy in the deposited genome sequences) for species “B”, which is shared with a species “A” strain, causes the erroneous attribution of its peptides to species “A”, just because of the differences in the degree of information available for the two related species.

Differently from most of the published studies regarding integration between metagenomics and metaproteomics, the 9MM assembled in this work included also two fungal species. This was to acknowledge the importance of *Fungi* as relevant members of microbial communities. Specifically, the great interest on commensal fungi and their key functions for health and disease is opening the way to the study of the so-called “mycobiome” [72], [73], [74], [75]. The data presented here highlight that further efforts are needed to optimize characterization of fungal species, and in particular to enable an efficient extraction of yeast DNA together with the more accessible bacterial DNA. As mentioned above, the metagenome-derived DB displayed an almost complete lack of eukaryotic sequences, thus impairing the identification of the corresponding peptides upon shotgun MS analysis. When considering only bacterial data, results attained using Meta-PA were comparable to those obtained with the remaining DBs (e.g., 3729 bacterial peptide identified with Meta-PA versus 4698 with SGA-PA and 4601 with TrEMBL-BFV). On the contrary, the exploitation of a proteogenomic approach can be useful to increase yeast metaproteome coverage (rather than for the bacterial counterpart), most likely in view of the presence of alternative or non-conventional splicing forms in eukaryotes [76]. It has also to be recognized that different bioinformatic strategies could have been alternatively used for genome sequence assembly, CDS finding, and gene annotation, especially to improve the quality of the 9MM-B metagenome draft which was not satisfactory (perhaps due to the extreme harshness of the extraction conditions used to improve yeast DNA yield). Therefore, we cannot rule out that the application of data analysis approaches different from the ones chosen in this work might have led to a higher metagenome, and consequently metaproteome, coverage.

Finally, it should be noted that the number of peptides (for metaproteomics), as well as the number of reads (for metagenomics), attributed to each of the 9MM microbes was far from being equal, although these were theoretically present in comparable amounts based on CFU counting. This might be explained by the fact that the nine microbial species exhibited significant differences in size and cell structure. In turn, such differences are in agreement with different amounts of proteins per cell between the nine microorganisms.

In conclusion, the results of this work confirm that DB selection is not a trivial issue in metaproteomics: data quality and quantity can vary dramatically depending on this factor. Based on our data, the following critical consideration and suggestion can be made: i) when possible, the parallel use of multiple DBs has to be encouraged, as different DB types can lead to highly complementary results; ii) the use of iterative metaproteomic searches with DBs of decreasing size, based on protein identification data obtained with relaxed FDR thresholds [66] or on taxonomic information obtained using generic DBs (as proposed in this work), can be key to achieve a wider metaproteome coverage; iii) especially when dealing with poorly characterized microbial community samples, metagenomics (and, in some cases, sequencing of individual genomes) can help investigate less characterized species; however, special care needs to be taken in metagenomic data processing to ensure an adequate quality of the derived DBs [30]; iv) software enabling LCA analysis of metaproteome data (namely, Unipept and MEGAN) can provide reliable results even at the species level, but proper filters with specific thresholds (e.g. based on the total number of taxon-specific peptides, such as the one proposed in this work) have to be set to reduce false positive attributions. On the whole, these data may be useful for all researchers dealing with microbiome characterization, and provide critical and concrete suggestions to improve reliability and analysis depth of metaproteomic results.

## Supporting Information

Data S1 (XLSX)

Data S2 (TIF)

Data S3 (XLSX)

Data S4 (XLSX)

Data S5 (XLSX)

Data S6 (XLSX)
