# Supplementary figures and images for: Evaluating the Impact of Different Sequence Databases on Metaproteome Analysis: Insights from a Lab-Assembled Microbial Mixture
Source: PLoS One. 2013 Dec 9;8(12):e82981. doi: 10.1371/journal.pone.0082981 (PMC3857319; doi:10.1371/journal.pone.0082981)

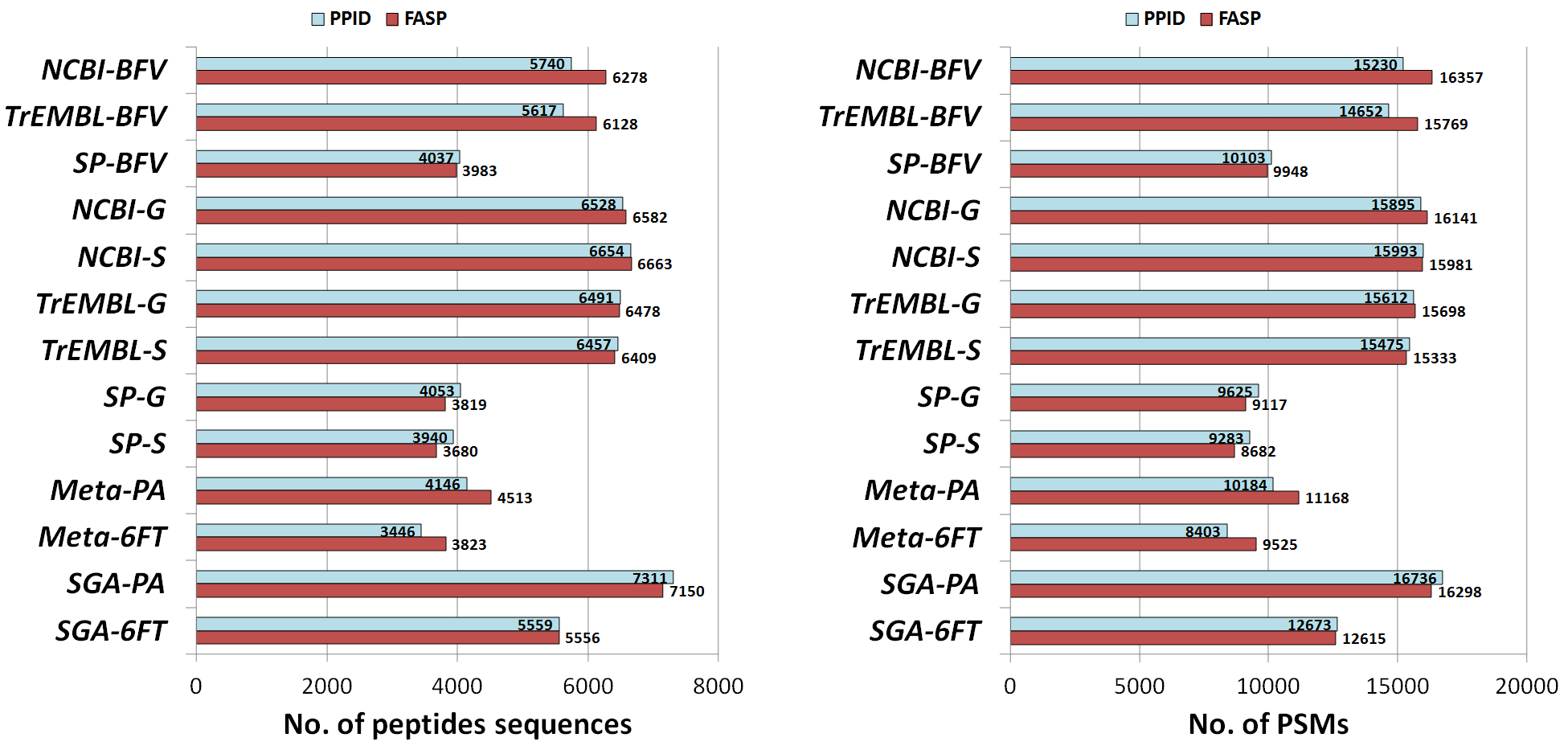

Supplement: Data S2 — (TIF) [file pone.0082981.s002.tif]
